# Effective Factors of Utilization of Inpatient, Outpatient, Diagnostic, and Pharmaceutical Health Services: A Systematic Review

**DOI:** 10.22086/gmj.v8i0.1236

**Published:** 2019-03-29

**Authors:** Solmaz Azimzadeh, Jafar Sadegh Tabrizi, Shirin Nosratnejad, Mostafa Farahbakhsh, Saber Azami Aghdash, Hossein Mashhadi Abdolahi

**Affiliations:** ^1^Iranian Center of Excellence in Health Management and Department of Health Service Management, School of Management and Medical Informatics, Tabriz University of Medical Sciences, Tabriz, Iran; ^2^Tabriz Health Services Management Research Center, Health Management and Safety Promotion Research Institute, Tabriz University of Medical Sciences, Tabriz, Iran; ^3^Department of Health Services Management, Faculty of Management and Medical Informatic, Tabriz University of Medical Sciences, Tabriz, Iran; ^4^Road Traffic Injury Research Center, Tabriz University of Medical Sciences, Tabriz, Iran

**Keywords:** Utilization, Health Services, Inpatient, Outpatient, Diagnostics, Pharmaceuticals

## Abstract

Utilization is one of the dimensions of equity in health systems. Identifying the factors affecting utilization of health services can be helpful for interventional purposes. This study systematically reviewed the factors affecting the utilization of inpatient, outpatient, diagnostic, and pharmaceutical services. This systematic review was conducted between 2016 and 2017. The search was performed using keywords based on MeSH in valid databases such as Scopus, Embase, ProQuest, ScienceDirect, PubMed, and Web of Science in the fields of title, abstract, and keyword. Related papers published from 2000 to 2017 were searched. First, the retrieved studies were screened and checked for quality; then, the useful data were extracted and analyzed. Out of the 1178 retrieved publications, 20 studies were included in the final analysis. The identified factors were categorized into 5 areas, including demographic (4 items), socioeconomic (13 items), health services–related (13 items), health status–related (7 items), and health insurance–related factors (2 items), and reported. The findings of this study can be a useful source and a comprehensive body of evidence on the utilization of health services. The results can be used by the policy makers and managers in designing interventions for changing the utilization patterns of health services.

## Introduction

Health is a matter of importance in today’s modern societies in such a way that we might conclude that improvements of health indicators lead to human and social development and then elevate the development status of the country [1]. Management of the health system requires evidence-informed decisions and some deep knowledge on the utilization of health services [2]. Today, health-related views have expanded and nonmedical determinants of health have gained special attention. These determinants, individually or by affecting others, seriously influence the health of people. Although health care results in the prevention of diseases, improvement of health, and treatment of illnesses, the socioeconomic status and the social determinants of health are highly influencing the health, illness, and need for medical care [3]. Reducing the inequalities in gender, races, and age groups is one of the main goals of the health policy makers [4]. Moreover, one dimension of equity is the utilization of health services [5]. The equitable access of all members of the society to health services leads to the improvement of health status in the whole population, and it prepares the requirements of growth and development [6]. As the effective factors on the utilization of health services are among the key determinants of community health, many researchers and health policy makers are interested in them [7]. Evidence shows that socioeconomic differences, by influencing the utilization rate of health services, lead to inequality in access to health care and thus an increase in the burden of diseases. Access is the first step for health services utilization and plays a main role in it [8]. Studies have shown that the utilization of health services is affected by many factors such as demographic factors, socioeconomic and cultural factors, health needs of the individuals, and access to the health facilities. On the other hand, poor socioeconomic conditions and lifestyle factors increase the burden of diseases, and resultantly, the need for health services [9-11]. Various models have been developed for understanding the pattern of health services utilization up to now. The model proposed by Andersen (2005) describes the health care utilization as a model of behavior. It considers the utilization of health services as a dependent factor of ability to use, barriers to use, and the need for the services. Furthermore, some people tend to use more health services than others, which can be predicted by some characteristics. Individuals with such characteristics of demographic factors, social factors, incentives, and beliefs are more likely to use the health services. In addition to the predictive factors of utilization, some other factors such as income, health insurance, availability, and access are necessary, without which the utilization would not occur [12]. The research on utilization of health services enables us to identify its facilitators and barriers and also to investigate the appropriateness of the volume and quality of the services [13]. The health services include a wide range of services, each of which is influenced by a number of other factors. Therefore, this study was conducted with the aim of identifying the effective factors on the utilization of inpatient, outpatient, diagnostic, and pharmaceutical health services.

## Materials and Methods

This systematic review was conducted between 2016 and 2017, and it was based on the Preferred Reporting Items for Systematic Reviews and Meta-Analyses guidelines.

### Data Sources

The existing scientific papers in English and Persian published between January 2000 and September 2017 were retrieved through searching English databases such as Scopus, Embase, ProQuest, ScienceDirect, PubMed, and Web of Science and also Persian databases such as SID and Magiran. Furthermore, the Google Scholar search engine was used for additional search of the related literature. The search keywords were “healthcare utilization,” “healthcare utilization,” “health service utilization” and “factors affect*,” “factors influenc*,” “affect* factors,” “influenc* factors,” “clinic/outpatient,” “hospitalization/inpatient,” “drug/medicine,” and “paraclinic/diagnos*.” The references of the related papers were also checked by hand for additional studies.

### Inclusion and Exclusion Criteria

There were 2 main inclusion criteria: (1) papers published between January 2000 and September 2017 and (2) papers investigating the effective factors of the utilization of inpatient, outpatient, diagnostics, and pharmaceutical health services. Meanwhile, there were 4 major exclusion criteria: (1) studies conducted before January 2000, (2) studies without a published full text, (3) studies published in languages other than Persian and English, and (4) papers studying specific populations such as immigrants or a specific disease such as AIDS or any other specific diseases.

### Screening

The retrieved corpus was pooled in EndNote X7 software produced by Thomson Reuters Company, and the duplicates were identified and removed. Then, the irrelevant papers were identified and excluded first by title screening and then by abstract screening. Finally, the full texts of the remaining papers were reviewed for eligibility according to the inclusion and exclusion criteria.

### Quality Appraisal

To assess the quality of the included papers, the Strengthening the Reporting of Observational Studies in Epidemiology checklist was used [14]. This checklist includes 22 items. The assessment was performed by 2 independent researchers. Each item had 1 score, and if the item had not passed, it got 0 score. In case of disagreement between the assessors about the score of the paper on a specific item, the case was discussed and resolved. Finally, those papers that gained 50% and more of the total possible score were included in the final analysis.

### Data Extraction

The required data were determined based on the study purpose and then extracted by one researcher and rechecked by another. The collected data were entered into an MS Excel spreadsheet in a table. The extraction table included information such as names of the authors, publication year, country, study type, sample size, data collection method, related findings, and the result of the quality assessment of the paper. To develop the table, the data related to 5 studies were extracted as pilot, and then the table was reviewed and revised.

### Data Analysis

Content analysis, which is a widely used qualitative research technique, was used in this study to analyze the extracted data. First, the influencing factors were identified from the content of the included papers. Then, the findings were coded and categorized as similar and related factors to be in the same category. The categories were named to describe the contents. Finally, the frequency of the codes was counted, and the results were presented in tables.

## Results

**Figure 1:**
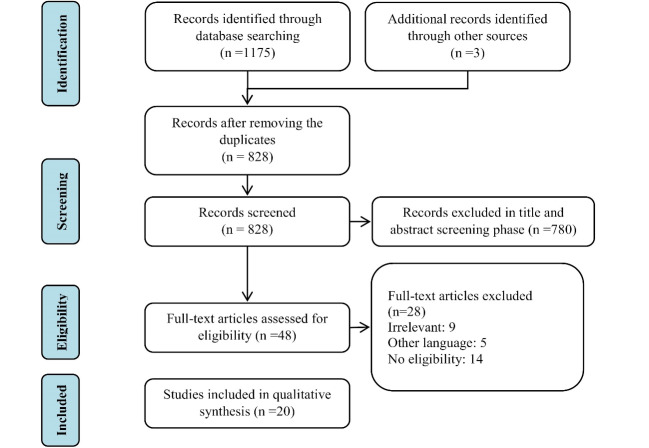
Search and selection process of the studies

Out of the total 1178 retrieved papers, 350 were duplicates that were excluded from the study. The remaining 828 records were first screened by title and then by abstract. Finally, after finishing the screening phase, 20 studies were included in the analysis. Figure-1 shows the search and selection process of the studies. The data were extracted from the final 20 included studies, and the affecting factors on the utilization of services were identified. The factors were divided into 2 groups of *positive effect* and *negative effect*. Table-1 shows the characteristics of the included studies and the main findings of each study as the factors affecting the utilization of health services. Out of the total 20 included studies, 12 were conducted in Asia and 8 were cross-sectional. The data used in 6 studies were secondary data. The health services were arranged in 4 groups of inpatient services, outpatient services, diagnostic procedures, and pharmaceutical services. Four studies had investigated the pharmaceutical services, 6 studies the diagnostic services, 8 studies the inpatient services, and 10 studies the outpatient services. A total of 45 factors were identified as having a positive effect on the utilization of health services and 8 factors as having a negative effect. The identified factors were classified into the following 5 categories:

1. Demographic factors (n=4), which included age, sex, marital status, and family size.
2. Socioeconomic factors (n=12), which included education, income level, employment, race, household expenditures, urban area, living place, using social media, health literacy, regular study, rented house, and the characteristics of family members.
3. Health service–related factors (n=13), which included physician visit, use of preventive care, medical technology growth, medical regulations,
4. hospitalization, length of hospital stay, grade of hospital, use of traditional medicine, physician’s qualifications, provider experience, access to health facilities, distance to health facilities, and regular care.
5. Factors related to the health status of individuals (n=8), which included chronic diseases, poor health condition, stressful life event, smoking, alcohol consumption, regular physical activity, days with disability, and inactivity.
6. Health insurance–related factors (n=2), which included insurance status and the insurance type.

Table-2 shows the summary of the identified effective factors on the utilization of health services.

## Discussion

This study reviewed the effective factors on the utilization of health services worldwide in the following 4 categories: pharmaceutical services, diagnostic procedures, inpatient services, and outpatient services. The identified factors were organized into 5 groups: demographic factors (4 factors), socioeconomic factors (13 factors), factors related to health services (13 factors), factors related to health status of the individuals (7 factors), and factors related to health insurance (2 factors). The determinants of health services utilization were vast and included a variety of factors that were related to the people and health care providers. The factors had been studied differently in various studies. Some studies had categorized the factors by the type of services, whereas other studies had categorized them by the type of clients. In this study, we tried to summarize the determinant factors of health services utilization in a simple and understandable way. Numerous studies have assessed the utilization of health services in different countries and in various populations to depict the demand for the services. Thus, the health system would be able to provide the services in a responsible manner to meet the health needs of the population. Findings of the studies on individual, social, and economic determinants of health services utilization indicate the needs of the societies for the health services. These findings can be used by the health system policy makers at the regional and national levels. For instance, studies that performed in China and Britain by the survey data found that similar socioeconomic groups have a similar utilization rate of the health services. Hence, based on these findings, they were looking for approaches to decrease the inequalities between the socioeconomic groups [15]. The results of the study showed that the demographic and socioeconomic factors were investigated more than other factors and were reported by more studies to have an effect on utilization of health services, in a way such that the demographic factors had been investigated in 15 studies and the socioeconomic factors in 16 studies out of the 20 included studies. Yet, some factors that correlate with health service utilization in a particular population on a particular group of health services may show no correlation in another population or another group of services. Even the effect of the factor might differ in different situations, for example, a particular factor may have a positive effect on the utilization of some services and simultaneously a negative effect on other services or in other regions or populations. Examples of this statement are discussed below. To conclude, the determinant factors of health services utilization are, to some extent, region-specific, and it is difficult to generalize them to all populations. In spite of this, findings of this review provided a basic understanding of the effective factors on the utilization of health services around the world. They can also be used as a list of potential factors for future studies. Some studies have mentioned education as a positive influencing factor on the utilization of health services [3, 12, 16, 17]. This means that more the education level of the individual, more the use of health services. However, 2 studies reported that by increasing the education level, the utilization rate of health services decreases [11, 18]. This might be justified by the assumption that people with more education have a better health status and they need health services less than others. A similar conflict exists about sex. Pappa *et al.* (2011) reported that women use more pharmaceuticals than men [18]. Sistrom *et al.* (2012) reported that women use more diagnostic services than men [19]. Tian *et al.* (2010) and Borhaninejad *et al.* (2015) reported that men use more inpatient services than women [20], whereas the study by Gong *et al.* (2016) found women using more inpatient services than men [21]. Such differences might be justified by cultural, social, and genetic differences of the studied populations. Moreover, Mendoza-Sassi (2003) and Nain (2013) reported a relatively higher use of outpatient services by women [11, 17]. Yet, the studies by Dunlop (2000) and Miltiades (2008) found that men use more outpatient services [10, 22]. It seems that the higher rate of utilization of health services by women is due to their higher sensitivity and attention to the health issues and preventive measures. Although household income was reported in some studies to have a positive effect on the utilization of health services [3, 9, 11, 12, 15, 23, 24], it was reported to have a negative effect in one study [25]. The study by Lee *et al.* (2011) indicated that the low-income people used more health services when they were covered by a type of health insurance. Moreover, some studies reported that those households that had high expenditures also had a high rate of utilizing inpatient services [21, 26]. However, the study by Nooraiee Motlagh *et al.* (2014) showed that those families who had high expenditures, such as a rented house, had a low rate of utilizing inpatient services [27]. It seems that it depends on other factors such as out-of-pocket payment and the insurance coverage. The nature of the household expenditure as an indicator is that the high expenditure in some cases means a high income; but in other cases, it may mean the low ability of the families to spend on health services, especially when they are not covered by any type of health insurance or the out-of-pocket payment is high. There was also a conflict regarding the role of smoking and alcohol consumption. Although the study by Dunlop *et al.* found smoking and alcohol to have a positive effect on utilizing outpatient services [12], Gong *et al.* reported that less smoking and alcohol consumption results in a higher use of inpatient and outpatient services [21]. This might be due to an incorrect identification of smokers and alcoholics in the conducted studies or the assumption that those people who had risky behaviors in the past had started to care more about their health. As smoking and alcohol are risk factors for many noncommunicable diseases, it can be assumed that many consumers have cardiovascular diseases, blood pressure, or other related diseases that oblige them to use more health services. Another possible reason might be that the smokers and alcoholics, due to concerns about their health, use more physician visits, screening, and diagnostic services. Regarding the people with chronic diseases, there was a unanimous agreement between almost all studies that these people had reported to have a higher utilization of all categories of health services [12, 16, 18, 19, 20, 22, 25, 26, 28, 29]. This is probably due to their higher need for health services. As expected, the health insurance coverage and having private health insurance were reported to have a positive effect on the utilization of health services [15, 17, 21, 23, 24, 30]. As health insurance provides better financial access to health services, those covered by a type of health insurance tend to use health services more than uninsured ones. The main limitation of this study was that it merely included the studies published in Persian and English. There might be some studies in other languages that would add to the findings of the study. However, the authors of this study used English as it is the formal language of most international scientific journals and Persian as it is the formal language of Iran.

## Conclusion

This study systematically reviewed the literature on utilization of pharmaceutical, diagnostic, inpatient, and outpatient health services. The identified factors that affect the utilization of health services were categorized as demographic, socioeconomic, health services–related, health status–related, and health insurance–related factors. Identifying the effective factors of utilizing health services can, first, depict the health needs of the population, and then, help the policy makers decide the best options to satisfy the needs. Such results can also help the policy makers in rational and evidence-informed decision making on planning the health services provision.

## Conflict of Interest

None declared.

**Table 1:** Characteristics of the Included Studies and Their Main Findings as the Affecting Factors On the Utilization of Health Services

**Author/year**	**Country**	**Scope**	**Study Population and Sample Size**	**Study Design**	**Data Collection Method**	**Factors**		**Quality Score (out of 22)**
						**Positive Effect**	**Negative Effect**	
Cifaldi (2001)	Canada	Pharmaceuticals	9 articles	Review	Paper review	Medical care utilization, health status, insurance coverage, income	-	16
Pappa *et al.* (2011)	Greece	Pharmaceuticals	968 patients	Cross-sectional	Interview	Increased age, education, visits to physician (4 times or more), poor physical and mental health, having at least one chronic disease, gender, married, living in urban area, employment status	-	18
Sistrom *et al.* (2012)	Massachusetts (USA)	Diagnostics	85277 patients, 148 physicians	Retrospective cohort study	Secondary data	**Patient factors:**race, health problem, visits to the linked PCP (preventive care physician), visits to specialists **physician factors:** years of experience, physician degree, clinic size	Provider experience, gender (male)	19
Quaas *et al.* (2014)	New York (USA)	Diagnostics	584 patients	Prospective observational study	Interview	Medicolegal liability Physician reassurance, Patient reassurance,	Cost	18
Lee *et al.* (2013)	Taiwan	Diagnostics	142123 patients	Survey	Secondary data	Physician visited, major illnesses, chronic disease, occupational diseases/injuries	-	15
Lee *et al*. (2011)	Taiwan	Diagnostics	241843 patients	Survey	Secondary data	Specialists visit, severe diseases, disabled subjects, chronic illnesses, length of stay	Income, urbanized	17
Siti Fatimah et al. (2015)	MALAYSIA	Diagnostics	234 patients	Cross-sectional	Questionnaire	Health knowledge, personal interest, marital status, general health problems	-	17
Tian et al. (2010)	Taiwan	Inpatient	1478 patients	Survey	Interview	Gender (males), health condition, specific chronic illnesses	Utilization of preventive care, economic hardship	20
Abenhai et al. (2008)	USA	Inpatient	45 630 patients	Retrospective cohort study	Secondary data	Median household income, insurance status (private insurance), race (Caucasians)	-	19
Ohta and Kronenfeld (2011)	USA	Inpatient	3409 patients	Survey	Secondary data	Age, race/ethnicity (Hispanic), insurance type, hospital-level variables	-	18
Mendoza-Sassi et a.l (2003)	Brazil	Outpatient	1260 patients	Cross-sectional study	Interview	Age groups (25-44 years and 65 years or over), marital status, stressful life events, regular source of care, health insurance, inactivity, potential serious symptom, chronic health problem, income	Education, gender (male)	19
Dunlop et al. (2000)	Canada	Outpatient	17, 626 patients	Survey	Interview	Number of health problems, perceived health status (poorer health status), regular care, marital status, income, education, physical inactivity, smoking, alcohol drinking	Gender (male)	19
Miltiades and Wu (2008)	China Boston (USA)	Outpatient	420 patients 177 family	Survey	Questionnaire	Chronic conditions, married, use of traditional Chinese medicine, poorer health status chronic conditions, health status, age, and social network, insurance status	-	18
Nain et al. (2013)	Malaysia	Outpatient	183 patients	Cross-sectional study	Interview	Higher education level, good quality of services, short distances between their residences and the health clinics	-	17
Gholami et al. (2015)	Iran	Outpatient	400 households	Cross-sectional	Interview	Gender of the head of household (male), marital status of the head of household, household size, education level of the head of household, job of the head of household, economic status of the household, regular study	Regular exercise	13
Wong et al. (2012)	Netherlands	Pharmaceuticals	10000 patients	Cross-sectional	Secondary data	Age +65		20
		Inpatient				Age (0-4 (newborn) and ages 80-84 in both and 30-34 years in women), growth in medical technology		
		Outpatient				Age +65	-	
Gong et al. (2016)	China	Inpatient	18246 patients	Longitudinal Study	Interview	Age +65, marital status, ethnicity, residents in urban areas, people with healthy lifestyles (such as not smoking or drinking, and having regular physical exercises), higher household expenditure, health insurance type, poor health conditions, physical disability, ADL (activities of daily living) limitations commercial insurance, education, gender (male)		20
		Outpatient				Age, marital statuses, ethnicity, people with healthy lifestyles (such as not smoking or drinking, with regular physical exercises), higher household expenditure, insurance type, poorer health status (physical & mental)		
Ebadifard Azar et al. (2011)	Iran	Pharmaceuticals	390 individuals, 130 households	Cross-sectional	Interview	Age of father, job of father, age of mother, education of father, education of mother, income, living area, number of children, health insurance type	-	-
		Diagnostics				Age of father, job of father, age of mother, job of mother, education of father, education of mother, household income, living area, number of children, health insurance type	-	
		Inpatient				Age of father, job of father, job of mother, education of father, education of mother, household income, living area, number of children, health insurance type	-	
		Outpatient				Age of father, job of father, age of mother, job of mother, education of father, education of mother, household income, living area, number of children, health insurance type	-	
Nooraiee Motlagh et al. (2014)	Iran	Inpatient	33000 households	Retrospective	Questionnaire	Households that have a member with chronic disease or elderly over 65 years or children under 5 years, households of higher income	Female head of the household, higher education of head of the household, rented house	15
		Outpatient				Households that have a member with chronic disease or elderly over 65 years or children under 5 years, households of higher income, female head of household, bigger family size, insurance coverage	-	
Borhani nejad et al. (2015)	Iran	Inpatient	600 individuals	Cross-sectional	Questionnaire	Age (age group 74 to 90 years), health insurance coverage, poor self-estimation of health condition	Gender (male)	15
		Outpatient				Age (age group 60 to 74), education (illiterates), married, health insurance coverage, poor self-estimation of health condition	-	

**Table 2:** Factors Affecting the Utilization of Health Services

**Health Services**	**Categories**				
	**Demographic Factors**	**Socioeconomic Factors**	**Factors Related to Health Status**	**Health Services-Related Factors**	**Health Insurance-Related factors**
Pharmaceuticals	Age, sex, marital status, household size	Education, income, employment, urban area, characteristics of household members	Poor health condition, chronic diseases	Physician visit, hospitalization	Having health insurance coverage, having private health insurance
Diagnostics	Age, sex, marital status	Education, income, employment, race, household expenditure, urban area, health literacy, characteristics of household members	Health status of the individual, having chronic disease	Physician visit, hospitalization, hospital length of stay, academic degree of physician, experience of the provider, access to providers, regulations	-
Inpatient	Age, sex, marital status	Education, income, employment, race, household expenditure, urban area, rented house, characteristics of household members	Health status of the individual, chronic disease, smoking, alcohol, regular physical activity	Using preventive care, medical technology growth, hospital grade	Insurance coverage, private insurance
Outpatient	Age, sex, marital status, household size	Education, income, employment, household expenditure, urban area, rented house, characteristics of household members	Health status of the individual, chronic disease, smoking, alcohol, regular physical activity, stressful life events	Use of traditional medicine, regular care, distance to clinic	Insurance coverage, private insurance
